# Sporadic Coexistence of Parathyroid Adenoma, Papillary Thyroid Carcinoma, Pheochromocytoma, and Cardiac Myxoma: A Multidisciplinary Approach to an Extremely Rare Tumor Constellation

**DOI:** 10.7759/cureus.84134

**Published:** 2025-05-14

**Authors:** Yukio Umeda, Shohei Mitta, Yukihiro Matsuno, Shoji Yoshikawa, Kenichiro Azuma

**Affiliations:** 1 Cardiovascular and Thoracic Surgery, Gifu Prefectural General Medical Center, Gifu, JPN; 2 Food and Nutritional Science, Toita Women's College, Tokyo, JPN

**Keywords:** cardiac myxoma, carney complex, hypercalcemia, multidisciplinary management, multiple endocrine neoplasia, papillary thyroid carcinoma, parathyroid adenoma, pheochromocytoma, sporadic tumors, surgical sequencing

## Abstract

A 74-year-old woman was referred for evaluation of asymptomatic hypercalcemia detected during a routine health examination. Laboratory testing confirmed primary hyperparathyroidism, with elevated serum calcium and intact parathyroid hormone (PTH) levels. Further diagnostic workup revealed a constellation of neoplasms: a parathyroid adenoma, papillary thyroid carcinoma, a right adrenal pheochromocytoma, a non-functional left adrenal cortical adenoma, and an incidental left atrial myxoma. There was no family history suggestive of hereditary tumor syndromes, and the patient declined genetic testing despite medical recommendation. A multidisciplinary team, including specialists in endocrinology, urology, otolaryngology, cardiology, cardiovascular surgery, and anesthesiology, collaborated to formulate a treatment strategy that minimized perioperative risks. Given the risk of hypertensive crisis, the right adrenal pheochromocytoma was resected first following adequate alpha-adrenergic blockade. This was followed by parathyroidectomy and left thyroid lobectomy with central neck dissection. Finally, to prevent potential embolic complications, the left atrial myxoma was excised via a transseptal approach under cardiopulmonary bypass. Histopathological examination confirmed the diagnoses of all tumors, with no evidence of malignant transformation or residual disease. This case illustrates a rare constellation of endocrine and cardiac tumors. Although the presentation raised clinical suspicion for hereditary syndromes such as multiple endocrine neoplasia (MEN) or Carney complex, the patient's advanced age, absence of mucocutaneous features, and negative family history favored a sporadic etiology. Nonetheless, the unusually delayed onset raises the possibility of a low-penetrance or as-yet-undescribed hereditary syndrome. Strategic surgical sequencing and interdisciplinary coordination were critical to ensuring patient safety and favorable clinical outcomes. Lifelong follow-up remains essential to monitor for recurrence and to reassess genetic risks as genomic technologies continue to evolve.

## Introduction

The coexistence of multiple endocrine tumors and cardiac myxoma in a single patient is exceedingly rare and typically raises suspicion for underlying hereditary syndromes such as multiple endocrine neoplasia (MEN) [1] or Carney complex [2]. These syndromes are characterized by distinct patterns of tumor development across endocrine and non-endocrine organs, often associated with identifiable genetic mutations. For example, MEN1 typically involves tumors of the parathyroid, pancreas, and pituitary, while MEN2 includes medullary thyroid carcinoma, pheochromocytoma, and hyperparathyroidism. Carney complex is characterized by cardiac myxomas, endocrine overactivity, and mucocutaneous pigmentation. However, not all patients who present with these tumor combinations meet the clinical or genetic criteria for the Carney complex syndromes. In sporadic cases, especially when genetic testing is declined or inconclusive, clinicians face significant diagnostic uncertainty.

Among the tumors involved, pheochromocytoma presents a particular perioperative risk due to catecholamine secretion, necessitating careful preoperative management and prioritization in surgical planning. Additionally, the incidental discovery of an asymptomatic cardiac myxoma poses a latent risk of embolic complications, warranting timely surgical intervention even in the absence of symptoms.

We report a unique case of a 74-year-old woman diagnosed with parathyroid adenoma, papillary thyroid carcinoma (PTC), right adrenal pheochromocytoma, and a left atrial myxoma, all identified during the diagnostic workup for hypercalcemia. This case highlights the complexity of managing multiple coexisting tumors, the challenges in differentiating sporadic occurrences from hereditary syndromes, and the critical role of multidisciplinary collaboration in achieving optimal patient outcomes.

## Case presentation

A 74-year-old woman was referred to our hospital for evaluation of hypercalcemia detected during a routine health checkup. Laboratory tests revealed elevated intact parathyroid hormone (PTH) at 163 pg/mL, indicating primary hyperparathyroidism. Her medical history included hypertension, impaired glucose tolerance, osteoporosis, thrombocytosis, previous surgery for uterine fibroids and ovarian cysts at age 57, and cholecystectomy for gallstones at age 58. Further investigations by the endocrinology department revealed multiple tumors.

Parathyroid tumor

**Figure 1:**
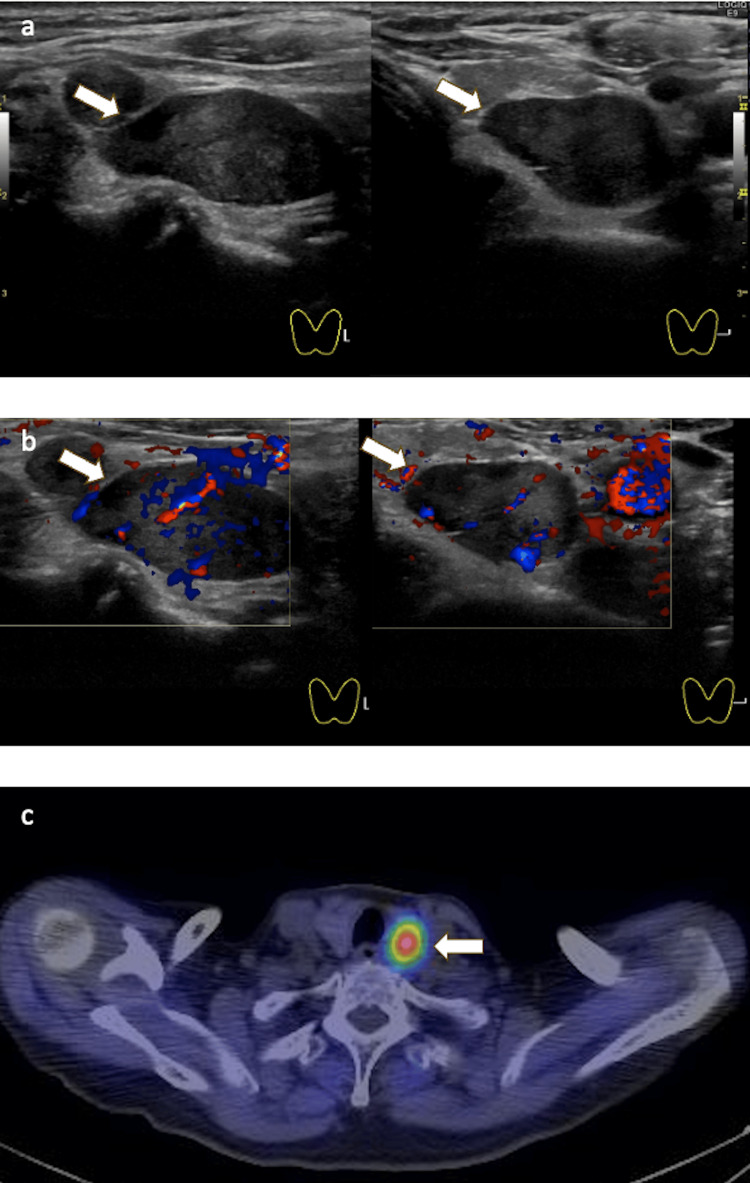
Parathyroid lesion. (a) A neck ultrasound revealed a well-demarcated hypoechoic mass measuring 26×21×12 mm lateral to the left thyroid lobe, with slightly heterogeneous internal echotexture. (b) Color Doppler ultrasound showed rich vascularity within the lesion. (c) MIBI scintigraphy demonstrated focal radiotracer uptake in the corresponding area. Arrows indicate the tumor and MIBI-avid region.

Laboratory evaluation showed hypercalcemia with a corrected calcium level of 13.1 mg/dL and elevated intact PTH (163 pg/mL), indicating primary hyperparathyroidism. A neck ultrasound revealed a well-demarcated hypoechoic mass measuring 26×21×12 mm located lateral to the left thyroid lobe, with slightly heterogeneous internal echotexture and rich vascularity (Figures 1a, 1b). Technetium-99m sestamibi (MIBI) scintigraphy demonstrated radiotracer uptake in the corresponding area, confirming a functional parathyroid adenoma (Figure 1c). No ectopic parathyroid tissue was detected in the delayed phase of the scan.

Thyroid nodules

**Figure 2:**
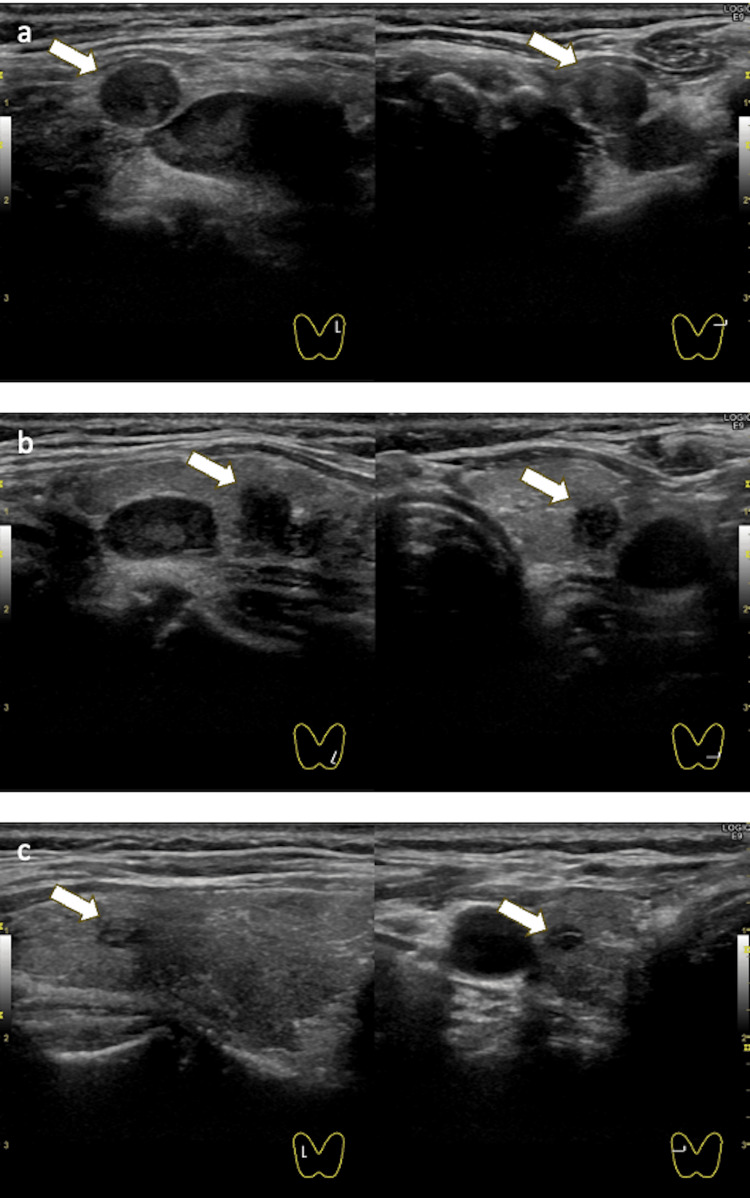
Thyroid nodules. (a) Hypoechoic nodule in the upper pole of the left thyroid lobe. (b) Hypoechoic nodule in the lower pole of the left thyroid lobe. (c) Cystic nodule in the right thyroid lobe with internal septations. Arrows indicate each nodule.

An ultrasound examination detected multiple nodules in both thyroid lobes. In the left lobe, two hypoechoic nodules measuring 9×7×7 mm and 5×5×5 mm were identified, both well-circumscribed, slightly heterogeneous internally, containing minor cystic components, and lacking detectable intra-nodular blood flow. In the right lobe, a 5×4×3 mm oval-shaped cystic nodule with septations and no vascularity was observed (Figures 2a-c). MIBI scintigraphy did not reveal abnormal uptake suggestive of medullary thyroid carcinoma. Fine-needle aspiration (FNA) of the left lobe nodule showed numerous clusters of follicular epithelial cells with granular chromatin, prominent nucleoli, nuclear enlargement, nuclear grooves, and intranuclear cytoplasmic inclusions, features suspicious for PTC. Thyroid function tests were within normal limits (thyroid-stimulating hormone (TSH) 1.40 µIU/mL, free T4 0.93 ng/dL, thyroglobulin 8.2 ng/mL).

Adrenal tumors

**Figure 3:**
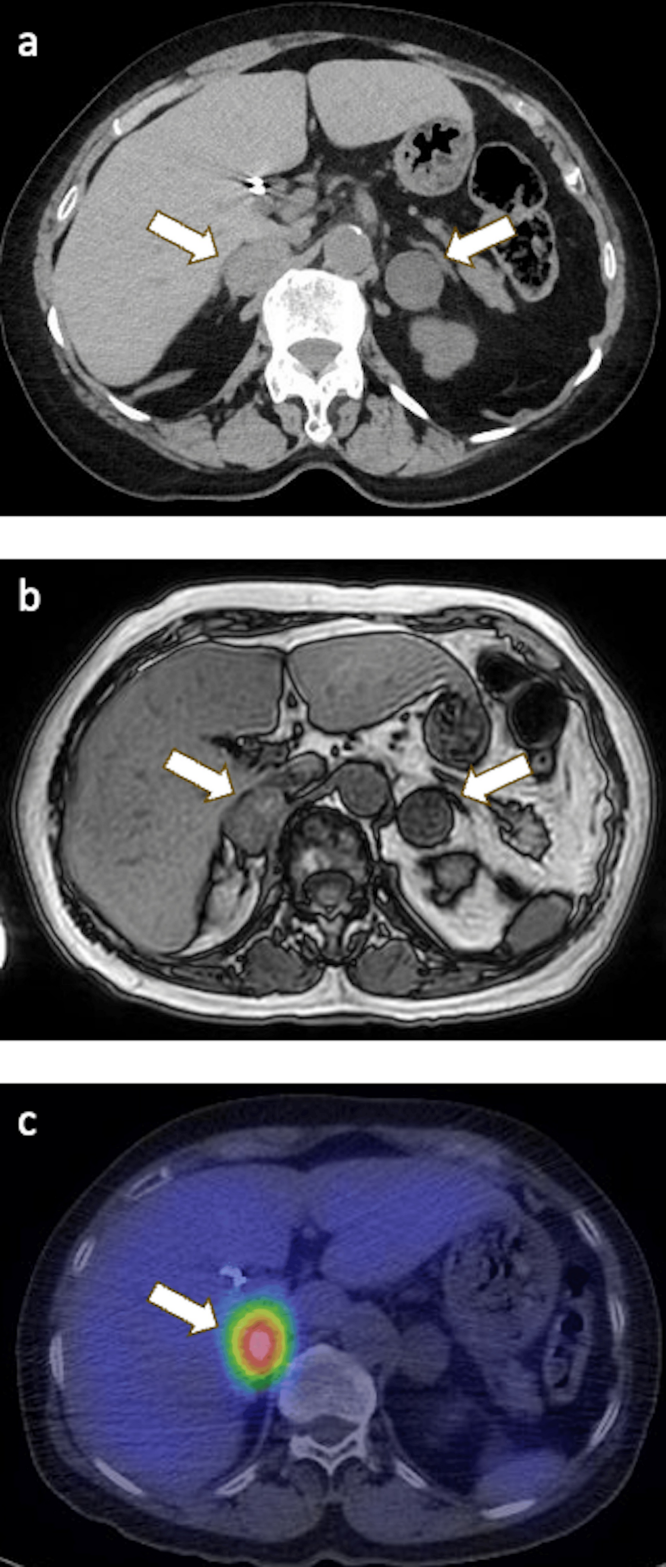
Adrenal lesions. (a) Abdominal CT showing a 32 mm right adrenal mass and a 26 mm left adrenal mass. (b) MRI depicting both adrenal masses. (c) MIBG scintigraphy revealing strong uptake in the right adrenal mass, with no uptake in the left. Arrows indicate each lesion.

Abdominal CT revealed a 32 mm right adrenal mass located adjacent to the inferior vena cava and liver, exhibiting high attenuation (~39 HU), atypical for a cortical adenoma. A 26 mm left adrenal mass showed fat density (-24 HU), suggestive of a cortical adenoma (Figure 3a). I-123 MIBG scintigraphy demonstrated strong uptake in the right adrenal mass, while no uptake was observed in the left mass (Figure 3c). Plasma catecholamine metabolites were elevated (metanephrine 187.7 pg/mL, normetanephrine 966.6 pg/mL), whereas adrenal cortical hormones remained within normal ranges (cortisol 5.9 µg/dL, aldosterone 6.6 pg/mL, dehydroepiandrosterone sulfate 44 µg/dL). These findings led to the diagnosis of right pheochromocytoma and left non-functional adrenal cortical adenoma.

Cardiac mass

**Figure 4:**
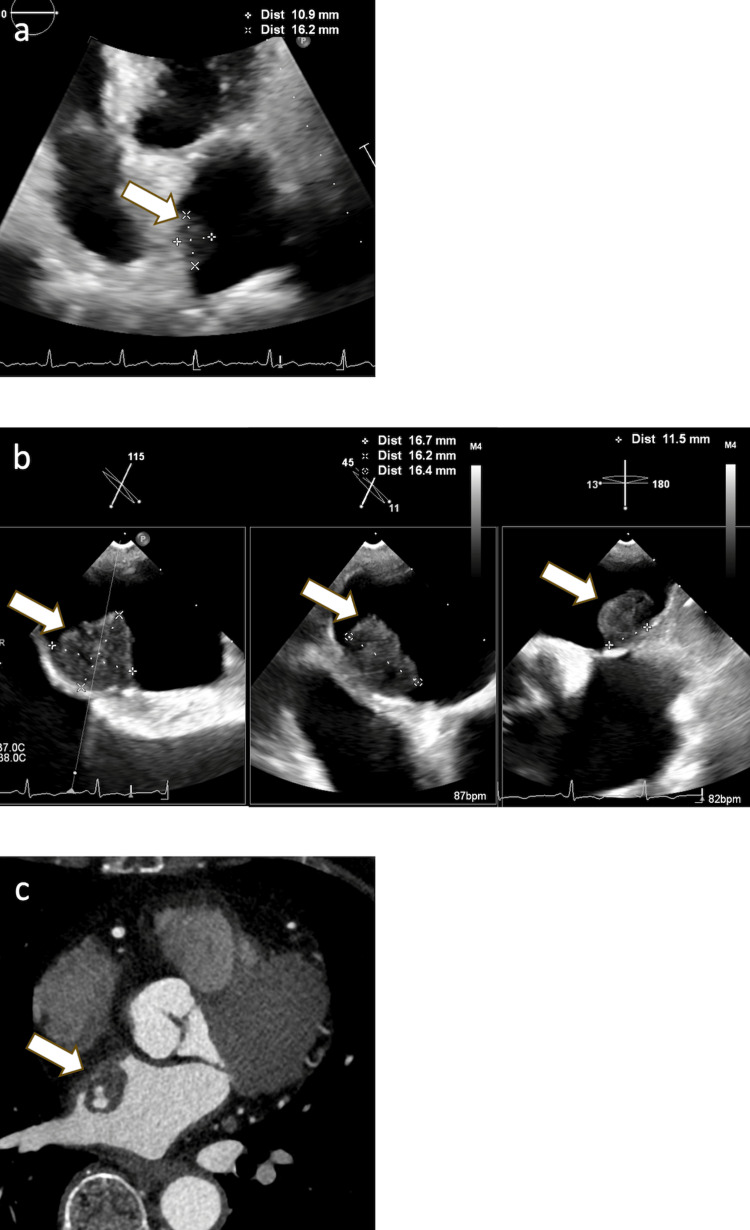
Left atrial mass. (a) Transthoracic echocardiography showed an 11×16 mm mass attached to the left atrial side of the interatrial septum. (b) Transesophageal echocardiography revealed a hypoechoic, irregularly shaped mass measuring 16.7×16.2×16.4 mm with a 10 mm stalk. (c) Contrast-enhanced CT demonstrated a 17×17 mm partially enhancing mass in the left atrium. Arrows indicate the mass in each modality.

Transthoracic echocardiography revealed an 11×16 mm mass attached to the left atrial side of the interatrial septum (Figure 4a). Transesophageal echocardiography further delineated a 16.7×16.2×16.4 mm hypoechoic mass with irregular margins, attached by a stalk approximately 10 mm wide (Figure 4b). Contrast-enhanced CT showed a 17×17 mm mass with partial contrast enhancement, attached to the left atrial side of the inter-atrial septum (Figure 4c).

Other findings

An abdominal CT revealed bilateral renal stones and a left renal cyst. The brain MRI showed mild enlargement of the pituitary gland (7.14 mm in height) relative to age, without evidence of adenoma. A summary of laboratory findings is shown in Table 1. Key laboratory findings included markedly elevated intact PTH at 163 pg/mL (reference: 10-65 pg/mL) and corrected calcium at 13.1 mg/dL (reference: 8.8-10.1 mg/dL), confirming primary hyperparathyroidism. Additionally, plasma normetanephrine was 966.6 pg/mL (reference: <506 pg/mL), consistent with pheochromocytoma. These values provided biochemical confirmation of the endocrine tumors identified radiologically.

**Table 1:** Summary of laboratory findings * Abnormal values. Reference ranges may vary depending on laboratory standards.

Parameters	Result	Reference Range	Unit
Sodium (Na)	139	138–145	mEq/L
Potassium (K)	4.6	3.6–4.8	mEq/L
Chloride (Cl)	104	101–108	mEq/L
Total Protein (TP)	7.8	6.6–8.1	g/dL
Albumin (ALB)	4.7	4.1–5.1	g/dL
Aspartate Aminotransferase (AST)	17	13–30	U/L
Alanine Aminotransferase (ALT)	14	7–23	U/L
Lactate Dehydrogenase (LDH)	171	124–222	U/L
Creatine Kinase (CK)	39	41–153	U/L
Alkaline Phosphatase (ALP)	110	38–113	U/L
Gamma-Glutamyl Transpeptidase (γ-GTP)	23	9–32	U/L
Total Bilirubin (T-Bil)	0.5	0.4–1.5	mg/dL
Blood Urea Nitrogen (BUN)	12	8–20	mg/dL
Creatinine (Cr)	0.52	0.46–0.79	mg/dL
Estimated Glomerular Filtration Rate (eGFR)	85.25	≥ 60	mL/min/1.73 m²
Glucose	116*	73–109	mg/dL
HbA1c (NGSP)	6.1*	4.9–6.0	%
Uric Acid (UA)	5.1	2.6–5.5	mg/dL
Triglycerides (TG)	173*	30–149	mg/dL
High-Density Lipoprotein Cholesterol (HDL-C)	59	40–90	mg/dL
Low-Density Lipoprotein Cholesterol (LDL-C)	122	70–139	mg/dL
White Blood Cell Count (WBC)	9.8	3.3–8.6	×10³/µL
Red Blood Cell Count (RBC)	4.1	3.86–4.92	×10⁶/µL
Hemoglobin (Hb)	11.5	11.6–14.8	g/dL
Hematocrit (Ht)	36.4	35.1–44.4	%
Platelets (Plt)	499	158–348	×10³/µL
Calcium	12.8*	8.8–10.1	mg/dL
Corrected Calcium	13.1*	8.8–10.1	mg/dL
Inorganic Phosphorus (P)	2.6*	2.7–4.6	mg/dL
Intact PTH	163*	10–65	pg/mL
Calcitonin	5.53*	≤ 3.91	pg/mL
1,25(OH)₂ Vitamin D	158*	20–60	pg/mL
25-OH Vitamin D	33	> 20	ng/mL
Plasma Metanephrine	187.7*	< 130	pg/mL
Plasma Normetanephrine	966.6*	< 506	pg/mL
Urinary Metanephrine	0.35*	0.04-0.18	mg/day
Urinary Normetanephrine	1.08*	0.10-0.28	mg/day
Plasma Renin Activity	0.8	-	ng/mL/hr
Adrenocorticotropic Hormone (ACTH)	7.9	7.2–63.6	pg/mL
Cortisol	5.9	4.5–21.1	µg/dL
Aldosterone	6.6	4–82.1	pg/mL
Dehydroepiandrosterone Sulfate (DHEA-S)	44	-	µg/dL
Thyroid-Stimulating Hormone (TSH)	1.4	0.61–4.23	µIU/mL
Free T4 (F-T4)	0.93	0.76–1.65	ng/dL
Thyroglobulin	8.2	< 33.7	ng/mL

Family and genetic evaluation

The patient had no family history of endocrine tumors, cardiac neoplasms, or features suggestive of hereditary syndromes such as MEN or Carney complex. Although genetic testing was recommended to evaluate potential syndromic associations, the patient declined genetic analysis.

Management and outcome

Preoperative Preparation

Initial medical management targeted hypercalcemia and hypertension secondary to pheochromocytoma. For primary hyperparathyroidism, treatment was initiated with eldecalcitol at 1-6 μg/day and elcatonin (40 U twice daily). Due to insufficient control, therapy was switched to a single dose of zoledronic acid (4 mg IV), resulting in a reduction of corrected serum calcium from 13.1 mg/dL to 10.6 mg/dL. For blood pressure control associated with pheochromocytoma, doxazosin was started at 1 mg/day and titrated up to 6 mg/day, successfully achieving a target blood pressure of 140/90 mmHg.

Surgical Strategy

A multidisciplinary team comprising endocrinology, urology, otolaryngology, anesthesiology, cardiology, cardiovascular surgery, and hepatobiliary surgery carefully planned the surgical sequence to minimize perioperative risks, particularly hypertensive crises and embolic events. The consensus was to proceed with staged surgeries in the following order: (1) right adrenalectomy for pheochromocytoma; (2) left parathyroidectomy and left thyroid lobectomy with central neck dissection; and (3) left atrial myxoma resection.

First stage (right adrenalectomy, March 2024): An open right adrenalectomy was performed due to tumor size, location, and proximity to critical structures. The 32 mm pheochromocytoma was located posterior to the inferior vena cava and adhered to the liver and surrounding tissues. Cardiovascular and hepatobiliary surgeons assisted with careful dissection to avoid vascular injury. The tumor was successfully resected without intraoperative hemodynamic instability or complications.

Second stage (left parathyroidectomy and left thyroid lobectomy, May 2024): Following stabilization, surgery for primary hyperparathyroidism and PTC was undertaken. A left parathyroidectomy and left thyroid lobectomy with central neck dissection were performed via a cervical approach. The procedure was uneventful, with no recurrent laryngeal nerve injury or postoperative hypocalcemia. Postoperative labs confirmed normalization of calcium and PTH levels. Pathology confirmed a functional parathyroid adenoma and PTC without lymph node metastasis.

Third stage (left atrial myxoma resection, April 2025): Although early surgical intervention is typically advised for cardiac myxomas due to the risk of embolic events, the timing of the third operation was primarily based on the patient's personal request to take a break after undergoing two consecutive surgeries. The patient expressed fatigue and a desire to recover fully before proceeding to the final stage. Notably, this delay was not due to endocrine-related perioperative concerns; as documented, blood pressure normalized soon after adrenalectomy, and both serum calcium and PTH levels returned to normal after the second surgery. All medications related to endocrine management (doxazosin, eldecalcitol, elcatonin, and zoledronic acid) were discontinued at that point. The multidisciplinary team deemed the short-term embolic risk manageable and respected the patient's preferences, scheduling the myxoma resection after sufficient recovery and shared decision-making.

**Figure 5:**
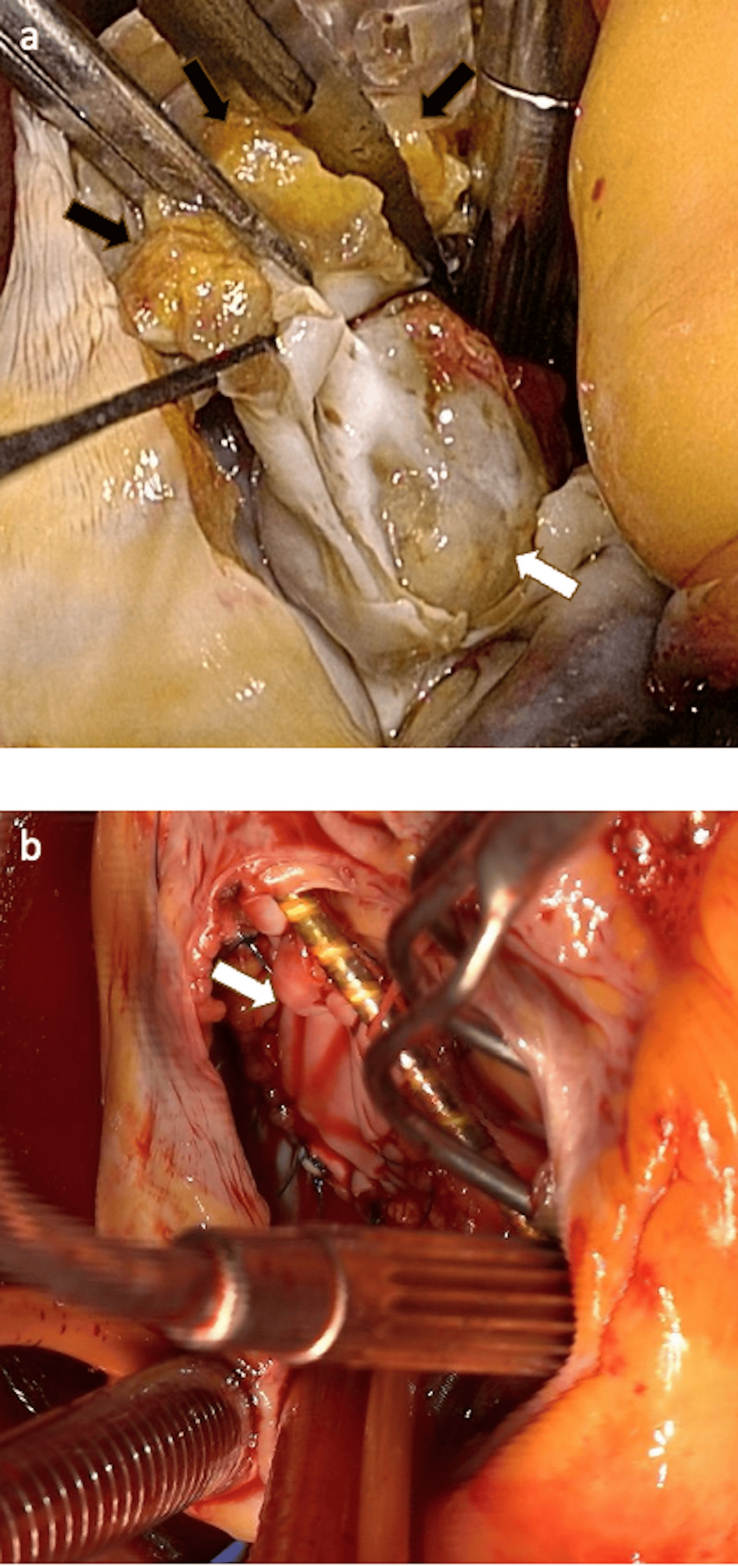
Intraoperative findings of cardiac tumor. (a) View from the right atrium after septal incision revealing the left atrial mass (white arrow). The black arrows indicate exposed adipose tissue at the resection margin. (b) Completion of double-layer bovine pericardial patch repair.

Under general anesthesia, a median sternotomy was performed, and cardiopulmonary bypass (CPB) was established. A right atriotomy provided access to the interatrial septum. A transseptal approach was used to excise the 17 mm myxoma en bloc with a margin of septal tissue. Although a myxoma was suspected, considering the patient's history of multiple neoplasms and the potential for a non-myxomatous tumor, a surgical margin of approximately 5 mm was deliberately secured. During resection, adipose tissue within the septum was exposed at the cranial and medial-lateral margins (Figure 5a). To ensure complete isolation of the left atrial cavity from the exposed adipose tissue, a double-layer bovine pericardial patch repair was employed (Figure 5b): (1) The first bovine pericardial patch was sutured to the left atrial endocardium and right atrial endocardium using interrupted mattress sutures; (2) a second patch was placed on the right atrial side and secured with continuous sutures incorporating the first patch and both atrial endocardia, ensuring complete isolation of the atrial cavities.

The patient was weaned from CPB uneventfully and was extubated on the first postoperative day. Postoperative echocardiography confirmed no residual mass or atrial septal defect.

Postoperative Course

The patient recovered well from all three surgeries without significant complications. Follow-up assessments showed stable calcium metabolism, normalized blood pressure without antihypertensive medication, and no signs of tumor recurrence or cardiac dysfunction.

Histopathological Findings

Adrenal tumors: The right adrenal mass was a well-circumscribed nodular tumor with a capsule continuous with the adrenal medulla. Histologically, tumor cells exhibited eosinophilic granular cytoplasm with amphophilic features, arranged in trabecular and nested patterns within a delicate vascular network. Immunohistochemistry was positive for synaptophysin and chromogranin A, with a Ki-67 labeling index of 2-5%. Scattered S100-positive sustentacular cells were observed. These findings were consistent with pheochromocytoma. Mitotic figures were noted at 2-3 per 10 high-power fields (HPF), with mild nuclear pleomorphism. While definitive vascular invasion was absent, focal infiltration into pericapsular adipose tissue was suspected. No tumor exposure at the surgical margin was identified. There was no evidence of adrenal medullary hyperplasia in the background adrenal tissue. The left adrenal mass was not resected, as it was diagnosed preoperatively as a non-functional cortical adenoma.

Parathyroid tumor: The resected mass was a well-demarcated tumor covered by a thin fibrous capsule. It consisted of relatively uniform tumor cells with eosinophilic cytoplasm, proliferating in alveolar and follicular patterns. Residual normal parathyroid tissue was observed at the periphery. There was no evidence of invasive growth, increased mitotic activity, or vascular invasion. These features were consistent with a diagnosis of parathyroid adenoma.

Thyroid nodules: Two nodules from the left thyroid lobe were examined. Nodule #1: A 0.8 cm lesion showing characteristic features of PTC with papillary architecture. There was minimal extrathyroidal extension without invasion into skeletal muscle. Nodule #2: A 0.4 cm lesion exhibiting follicular and papillary growth patterns, also consistent with PTC. No extrathyroidal extension was observed. The relationship between the two nodules was considered either intraglandular metastasis from Nodule #1 or synchronous multifocal disease. The final pathological staging was papillary carcinoma, pT1a(m).

Left atrial mass: A broad-based, polypoid lesion was observed, composed of a myxoid stroma containing sparsely distributed spindle- to stellate-shaped cells. These cells proliferate in isolated and loosely arranged patterns. The background shows evidence of hemorrhage, fibrin deposition, hemosiderin-laden macrophages, and prominent vascular proliferation.

Immunohistochemical staining revealed that the tumor cells were calretinin positive, CD34 positive, CD31 positive, EMA weakly positive, ​​α-SMA weakly positive, pan-cytokeratin negative, and desmin negative. These histological and immunohistochemical features were consistent with a cardiac myxoma. No evidence of tumor infiltration was observed at the surgical resection margins.

## Discussion

The simultaneous occurrence of parathyroid adenoma, PTC, pheochromocytoma, and cardiac myxoma in a single patient is exceedingly rare and strongly suggests an underlying hereditary syndrome, such as MEN types 1 or 2 or Carney complex. MEN1 is characterized by tumors of the parathyroid, entero-pancreatic, and anterior pituitary glands, with penetrance approaching 95% by the sixth decade of life, whereas MEN2 typically includes medullary thyroid carcinoma, pheochromocytoma, and hyperparathyroidism [1]. Carney complex features cardiac myxomas, endocrine overactivity, and spotty mucocutaneous pigmentation [2]. In contrast, our patient presented with papillary rather than medullary thyroid carcinoma and lacked mucocutaneous features, making classic MEN2 and Carney complex less likely.

Despite medical advice, the patient declined genetic testing, and no family history of endocrine or cardiac tumors was present. This case was discovered at age 73, significantly older than the usual onset range (10-40 years) for MEN1 and Carney complex. Nonetheless, late-onset manifestations of MEN1, such as asymptomatic pancreatic neuroendocrine tumors in patients over 70 years old, have been documented [3], and low-penetrance *RET* mutations (e.g., K666N) have been associated with delayed MEN2 diagnoses and milder phenotypes [4].

Clinicians encountering patients with apparently sporadic endocrine tumors, particularly when multiple organs are involved, should remain alert to subtle systemic findings. In our case, the combination of asymptomatic hypercalcemia, a catecholamine-secreting adrenal tumor, incidental cardiac mass, and mild pituitary enlargement, despite the absence of mucocutaneous signs or family history, should prompt consideration of a syndromic origin. Such presentations, though rare, may reflect low-penetrance or late-onset variants and warrant coordinated evaluation and follow-up.

Hence, although the current diagnosis is sporadic, the case may belong to a novel disease spectrum with low penetrance and delayed onset. Emerging evidence from germline whole genome sequencing (WGS) in adults with multiple primary tumors highlights that broad genomic assessment can uncover pathogenic or likely pathogenic variants not detected by standard multigene panel testing [5]. Such unusual tumor constellations, even when sporadic, may help prioritize patients for comprehensive genomic screening and improve recognition of low-penetrance hereditary syndromes.

Management of such a constellation demands a multidisciplinary team (MDT) approach to optimize outcomes. Our MDT included specialists from endocrinology, anesthesiology, otolaryngology, urology, cardiology, and general and cardiovascular surgery. Surgical resection began with the pheochromocytoma, in accordance with Endocrine Society guidelines recommending adrenalectomy before other procedures to avoid intraoperative hypertensive crises; adequate preoperative alpha-adrenergic blockade and cardiovascular monitoring ensured perioperative stability [6]. Subsequently, thyroid carcinoma and parathyroid adenoma were treated with cervical surgery, with careful recurrent laryngeal nerve preservation, and postoperative normalization of serum calcium and PTH levels confirmed successful parathyroid resection [7].

The incidental detection of a left atrial myxoma during preoperative cardiac evaluation highlights the importance of comprehensive assessment in systemic neoplastic diseases. Cardiac myxomas are the most common primary cardiac tumors but can remain clinically silent until catastrophic events such as embolism or sudden cardiac death occur. Early and complete surgical excision is advised even in asymptomatic patients to prevent such complications [8,9]. In cases associated with Carney complex, long-term surveillance for recurrent myxomas is paramount, given a recurrence rate of up to 22% [10].

Follow-up should be lifelong due to the possibility of delayed or evolving features of MEN or Carney complex. This includes (1) monitoring for tumor recurrence or regrowth, particularly for parathyroid hyperplasia and thyroid carcinoma; (2) surveillance of residual or latent neoplasms such as nodules in the residual thyroid, adrenal cortical adenomas, and pituitary lesions, both present in our case; and (3) periodic reassessment of genetic testing candidacy, especially with evolving technologies and guidelines.

The follow-up protocol comprises (1) annual biochemical screening (PTH, calcium, TSH, free T4, metanephrines, cortisol, ACTH, IGF-1, and prolactin); (2) targeted imaging (thyroid ultrasound, echocardiography, and adrenal and pituitary MRI); (3) patient education (symptom tracking for catecholamine excess, hypocalcemia, or embolic signs); and (4) family member counseling if new findings emerge or the patient consents to future genetic testing [1,11].

## Conclusions

This case illustrates the diagnostic and therapeutic complexity posed by the coexistence of multiple endocrine and cardiac tumors in a patient lacking confirmed hereditary syndromes. Despite clinical features suggestive of MEN or Carney complex, the absence of definitive genetic or phenotypic evidence required an individualized, risk-based management plan. The surgical sequence, starting with pheochromocytoma resection, followed by parathyroid adenoma, PTC, and finally cardiac myxoma, was critical to minimizing perioperative complications and optimizing patient safety.

Long-term, multidisciplinary follow-up remains essential, particularly in light of residual lesions such as a left adrenal cortical adenoma, thyroid nodule, and mild pituitary enlargement. Even in apparently sporadic presentations, the constellation of tumors and their late onset may signal a previously unrecognized hereditary syndrome with low penetrance. Continued surveillance and periodic re-evaluation using evolving genomic technologies may eventually identify novel pathogenic variants, enhancing understanding and management of such rare, complex tumor syndromes.
